# Organic Learning Gardens in Higher Education: Do They Improve Kindergarten Pre-service Teachers’ Connectedness to and Conception of Nature?

**DOI:** 10.3389/fpsyg.2020.00282

**Published:** 2020-03-10

**Authors:** Raquel Pérez-López, Marcia Eugenio-Gozalbo, Daniel Zuazagoitia, Aritz Ruiz-González

**Affiliations:** ^1^Department of Research and Psychology in Education, School of Education, Complutense University of Madrid, Madrid, Spain; ^2^Department of Experimental and Social Sciences, and Mathematics Education, Faculty of Education of Soria, University of Valladolid, Soria, Spain; ^3^Department of Didactic of Mathematics and Experimental Sciences, Faculty of Education and Sport, University of the Basque Country (UPV/EHU), Vitoria-Gasteiz, Spain

**Keywords:** nature, organic learning gardens, environmental concerns, pre-service teachers, connectedness to nature, phenomenography

## Abstract

Studies have shed light on the idea that people who have experiences in natural settings might be more aware of the environment. Learning gardens, as outdoor contexts, might contribute to the development of students’ affective relations toward nature, pro-environmental attitudes, and protective actions; neverthless, these aspects begging to be explored. This preliminary research investigates the impact that the use of organic gardens to teach natural sciences at university has on kindergarten pre-service teachers’ (KPST) connectedness to and conceptions of nature. The research follows a pre-/post-design and it uses a mixed methods approach. A total of 74 students completed four quantitative scales (INS, CCC, LCN, and NR-6), and 66 of them an open question about the concept of nature. After the garden experience, students scored higher in all the scales, nevertheless the change was significant only for INS and CCC. The phenomenographic analysis evidenced an initial predominant static and non-social concept of nature, biased toward the most obvious biological elements. After the garden-based learning experience, more informed conceptions of nature – including notions of complexity and systemic character – increased from 7 to 19%; however, statistical comparison was not significant. In spite of the absence of concluding results, further research is required to assess the role that learning gardens may play regarding connectedness to nature and pro-environmental behaviors.

## Introduction

The existence of a global environmental crisis is scientifically established (Lewis and Maslin, 2015); if current world’s population truly cares about future generations, the preservation of the planet appears as an obligation (Röckstrom and Karlberg, 2010). Thus, environmental education and sustainability education play a fundamental role in training citizens that are more aware of global change, and more environmentally responsible (Novo, 2017). Research supports that experiences in nature relate to the development of affective relations toward nature, pro-environmental attitudes, and protective actions (Chawla, 2009; Collado et al., 2013; Zelenski et al., 2015; Evans et al., 2018). However, contact with nature is becoming more infrequent in an increasingly urbanized world where most of the population is urban (World Watch Institute [WWI], 2016), in such a way that access to natural settings is not always possible, particularly for young people (International Union for Conservation of Nature [IUCN], 2016). The loss of human–nature interactions was reported 20 years ago and it is increasing, resulting in both the diminution of a range of benefits related to health and wellbeing, and in positive emotions, attitudes, and behaviors toward the environment (Soga and Gaston, 2016).

Since “direct experience of nature plays a significant, vital, and perhaps irreplaceable role in affective, cognitive, and evaluative development” (Kahn and Kellert, 2002, p.139), the need to increase education in nature or to naturalize school environments has been emphasized by International Union for Conservation of Nature [IUCN] (2016). There is empirical evidence of how outdoor classrooms increases wellbeing and boost subsequent classroom engagement (Kuo et al., 2018; Largo-Wight et al., 2018), and on the impacts of greening schoolyards on children’s health and wellbeing (Dyment and Reid, 2005; Johnson, 2007; Kelz et al., 2013; Dijk-Wesselius et al., 2018). Similarly, learning gardens are expected to allow children gaining outdoor learning experiences (Williams and Dixon, 2013; Sanders et al., 2018; Zelenika et al., 2018), and an incipient research shows their impacts on health (Dyg and Wistoft, 2018), including university students (Retzlaff-Fürst, 2016).

In Spain, the number of learning gardens is growing at primary and middle schools, and they are also being used as a context for natural sciences teaching in Higher Education, particularly for initial teacher training. Thus, University Organic Learning Gardens (UOLGs) allow students to experience sustainable land practices and encourage them to become aware of the need for nature conservation from cognitive, procedural, and affective dimensions (Eugenio and Aragón, 2016; Eugenio et al., 2018; Eugenio-Gozalbo et al., 2020). Pre-service teachers might become a key to spread knowledge and transfer pro-environmental values and skills to forthcoming generations, since whenever teachers perceive that implementing outdoor experiences result in school improvement (Sahrakhiz, 2017) and that support is given to them to further integrate the green schoolyard as a learning environment (Dijk-Wesselius et al., 2020).

Regarding the cognitive dimension, *Nature*, this is a widely used concept in both academic and daily languages, which has gained complexity over the time (Sharma and Buxton, 2018). Research highlights that students at different educational stages treat *nature* as synonymous of *environment*, and hold a range of *conceptions of nature*, from very simplistic (place for animals and plants to live), to more complex (dynamic domain with a diversity of biotic and abiotic elements, including humans, in relationships of mutual interdependence) (Loughland et al., 2002; Payne et al., 2014). In spite of this, students develop distinct perceptions, attitudes, and values about the natural world and the role people play in it, and they show inclination to protect and treat it with respect (Sharma and Buxton, 2018).

In this preliminary research, we aim to assess whether or not the use of UOLGs as learning contexts from where teaching natural sciences may influence cognitive and emotional dimensions of students’ relation with nature. Concretely, we will evaluate effects of compulsory natural sciences programs on connectedness to and the concept of nature in kindergarten pre-service teachers (KPST).

## Materials and Methods

### Participants

A total of 74 KPST participated in this study enrolled in one of the two compulsory natural sciences programs that took place during a semester and used an UOLG as a main setting for practical science lectures. The two programs corresponded to two equivalent subjects of the Degrees in Pre-School Teacher Training in two universities. Both subjects met the official scientific curriculum for pre-school education and use an university garden that constitues living laboratory where learning and experimenting with living beings and processes. Garden facilities are similar and conformed by a cultivate area, a tool house, and composting drawers. Students at both programs work in groups on gardening tasks for 2-h sessions a total of about six or seven times during the course. In class sessions, a total 44 participants took part in Program 1 (University of the Basque Country) and 30 enrolled in Program 2 (University of Valladolid). From all participants, 67 were female and 7 were male (*M*_age_ = 22 years; *SD* = 2.1).

### Measures

This research follows a mixed methodology to explore connections with the natural environment among KPST. As Creswell (2014) suggests, this procedure enables to deeper understand research question; in this regard, quantitative data would be better explained with qualitative ones. In this study, participants completed a questionnaire composed of several Likert scales aiming to measure cognitive, emotional, and attitudinal aspects of their connection to nature. Concretely, the whole sample filled up seven-point Likert scales: *Inclusion of Nature in Self-Scale* (INS) (Schultz, 2001), the *Love and Care for Nature Scale* (LCN) (Perkins, 2010), the *Nature Relatedness* (NR-6 Spanish version) (Pasca García, 2019), and a five-point Likert three-item scale about Climate Change Concerns (CCC)^1^. Additionally, an open question was used to explore the cognitive dimension: *Imagine you are the teacher of Year-9 class and you need to explain what nature is. Please write down the way you would explain it to your students.* All measures were completed at the beginning and at the end of the science programs.

### Data Analysis

Firstly, several mean comparison analyses were conducted with SPSS 20.0 software for all the quantitative scales (*N* = 74). Secondly, the open question was analyzed basing on a phenomenographic approach (Marton, 1988, 2015) (*N* = 66 due to missing values). A final system of nine hierarchic categories of growing complexity was defined enclosing the whole range of KPST’s *conceptions of Nature*. The degree of agreement reached between two researchers regarding allocation of each nature definition (Cohen’s Kappa reliability coefficient average 0.92) fell within the suggested range. A detailed description of the phenomenographic procedure is enclosed in the Supplementary Material. Finally, using the nine categories, a Wilcoxon signed ranks test was done to compare the complexity of the definitions before and after the intervention.

## Results

### Quantitative Data Analyses: Connectedness to Nature

For all the scales, participants considerably agreed, and higher values are found on the CCC scale (Tables 1, 2). The reliability analyses showed good adequacy for LCN and NR-6: Cronbach’s alpha was >0.8, meaning that these two scales have high consistency and they are adequate to measure connectedness to nature. Nevertheless, the reliability analysis for the CCC scale showed a Cronbach’s α of 0.66, indicating that the instrument is acceptable (Nunnally and Bernstein, 1994). Additional, several factor analyses were run resulting in one dimension scales (Tables 1, 2).

**TABLE 1:** Statistics, internal consistency for the four scales, and total variance explained for pre-test measures (*N* = 74).

	**Pre-test**				
**Scale**	**Number**	***M***	**SD**	**Cronbach’s**	**Total variance**
	**of items**			**α**	**explained**
INS	1	4.82	1.502	–	–
LCN	14	5.44	0.929	0.97	67.9
NR-6	6	5.03	0.988	0.88	62.6
CCC	3	4.23	0.555	0.66	60.3

*INS, Inclusion of Nature in Self scale; LCN, Love and Care for Nature scale; NR-6, Nature Relatedness scale; CCC, five-point Likert three-item scale about Climate Change Concerns.*

**TABLE 2:** Statistics, internal consistency for the four scales, and total variance explained for post-test measures (*N* = 74).

	**Pre-test**				
**Scale**	**Number**	***M***	**SD**	**Cronbach’s**	**Total variance**
	**of items**			**α**	**explained**
INS	1	5.30	1.331	–	–
LCN	14	5.51	0.918	0.97	69.2
NR-6	6	5.13	1.040	0.92	72.2
CCC	3	4.38	0.482	0.62	58.5

**FIGURE 1:**
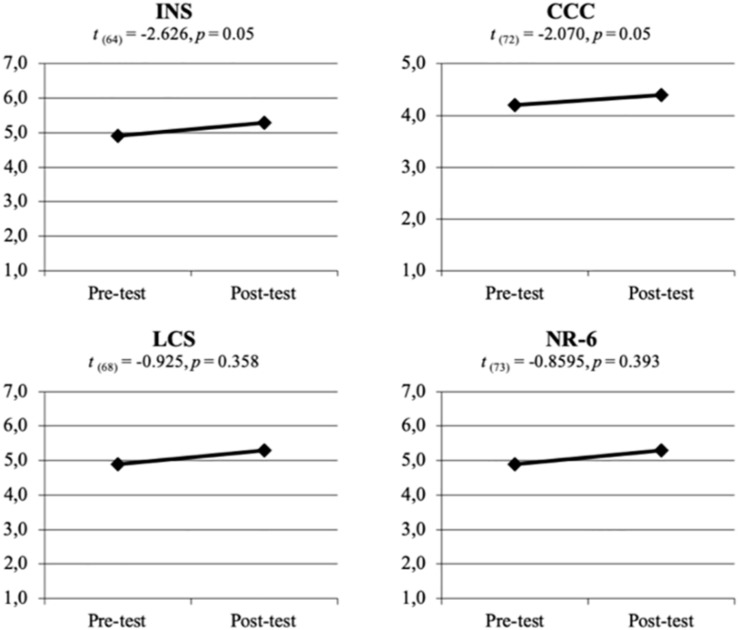
Comparison of pre-/post-intervention for INS, CCC, LCN, and NR-6.

In order to identify differences between programs, an ANOVA was conducted, and non-significant differences were found. Therefore, the comparisons before and after the intervention using OLGs was conducted with then whole sample. Comparisons pre/post showed significant differences for the INS (*t*_(__64__)_ = −2.626, *p* = 0.05) and the CCC (*t*_(__72__)_ = −2.070, *p* = 0.05) scales, but not for the LCN and NR-6 scales (Figure 1).

### Qualitative Data Analyses: Conceptions of Nature

Kindergarten pre-service teachers’ *conceptions of nature* were classified into nine categories corresponding to qualitatively different visions. Such categories were arranged to show the most important qualitative differences between conceptions, and from the least to the most inclusive and informed view (Table 3).

**TABLE 3:** Categorization of the main KPST’s conceptions about nature that were unveiled by means of phenomenographic analyses.

			**Pre % (*n*)**	**Post % (*n*)**
Static	Pristine		**19.7 (13)**	**21.2 (14)**
		C1-Bio(or)Physical pristine	9.1 (6)	4.5 (3)
		C2-Biophysical pristine	10.6 (7)	16.7 (11)
	Non-pristine		**42.4 (28)**	**37.9 (25)**
		C3-Bio(or)Physical	18.2 (12)	21.2 (14)
		C4-Biophysical	13.6 (9)	10.6 (7)
		C5-Biophysical diverse	10.6 (7)	6.1 (4)
Dynamic	Only human-nature interactions		**30.3 (20)**	**21.2 (14)**
		C6-Utilitarian	10.6 (7)	7.6 (5)
		C7-Preservation	6.1 (4)	6.1 (4)
		C8-Utilitarian + preservation	13.6 (9)	7.6 (5)
	Interactions among components of a complex system		**7.6 (5)**	**19.7 (13)**
		C9-Systemic	7.6 (5)	19.7 (13)

*KPSTs, kindergarten pre-service teachers. Bold values represent subcategories totals.*

Kindergarten pre-service teachers who described *nature* using *conceptions 1 to 5* understand it as a *static entity*, exclusively enlisting various bio and/or physical elements. From these static conceptions, some referred to *nature* as untouched or unmodified by humans (*static pristine conceptions of nature*: C1 and C2), while others did not explicitly acknowledge this aspect (*static non-pristine conceptions*: C3–C5). Finally, some of these students acknowledged the change and diversity of nature (C5).

Kindergarten pre-service teacher who described *nature* using *conceptions 6 to 9* go beyond and describe it as a *dynamic entity*. From these dynamic conceptions, some focused exclusively on value-oriented human to nature interactions (*utilitarian*-C6, *preservation*-C7, or *utilitarian* + *preservation*-C8), whereas others showed a more informed ecological view (*systemic*-C9).

Kindergarten pre-service teacher who showed the most comprehensive *conception of nature* (C9) consider it as a series of relations between different earth systems, beyond the human-to-nature interactions. This view was systemic and complex, at least partially, and it is the most aligned with contemporary *scientific conceptions*.

A detailed description of the categories and examples of each are provided as Supplementary Material.

Initial results outline a predominant *conception of nature* which was not only static (62.1%), but also non-social and biased toward the most obvious elements. Concretely, only around 14% of KPST explicitly enclose humans into *nature*. The elements that are mentioned mainly correspond to the biosphere (“plants and animals”), while those corresponding to other earth systems are anecdotal, such as the references to the hydrosphere, the geosphere, or the atmosphere. Final results showed some changes, therefore, whereas the percentage of static views reduced the dynamic views increased (±3%). Outstandingly, the frequency of the most comprehensive *conception of nature* (C9) increased in 2.6 times.

In order to compare the conceptions of nature before and after the intervention, a Wilcoxon signed ranks test revealed non-significant differences pre/post when considering the complete system of nine categories (*z* = −0.734, *p* = 0.463).

**FIGURE 2:**
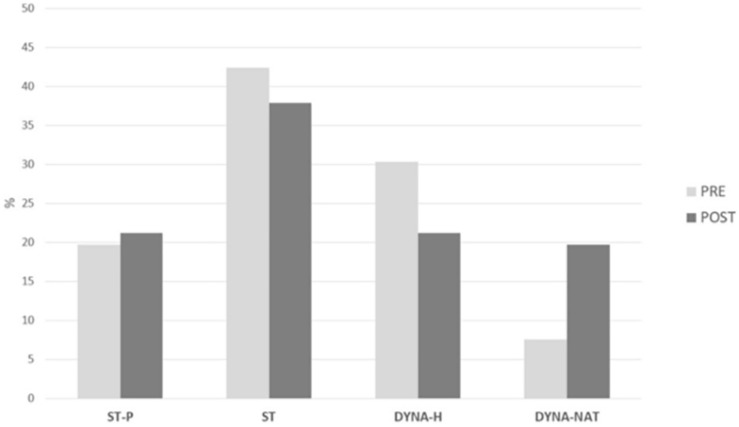
Frequency (%) of KPST’s conceptions of nature before (in gray) and after (in black) the educational experience at the OLG.

Figure 2 shows the frequency of KPST’s *conceptions of nature* as grouped according to four main categories: *Static pristine* (ST-P) (C1 and C2); *static non-pristine* (ST) (C3–C5); *dynamic only human–nature interactions* (DYNA-H) (C6–C8); and finally, *dynamic interactions among components of a complex system* (DYN-NAT) (C9).

## Discussion

This preliminary study aims to assess the impact that using UOLGs for practical science lessons may have on the emotional and cognitive dimensions of KPST’s relation with nature. Research studying educational experiences in natural environments uses quantitative (Kuo et al., 2018; Largo-Wight et al., 2018) or qualitative methods (Williams and Dixon, 2013), we follow a mixed methods approach, including quantitative analyses and a phenomenographic exploration to better understand the impact of the interventions. Regarding connectedness to nature, KPST considered themselves as relatively connected to nature, and their score values placed around five, higher that those found in other studies with Spanish undergraduates (Olivos and Aragonés, 2011; Amérigo et al., 2012). After taking part in the program, scores for the four scales increased, nonetheless significant differences were only identified for the INS and the CCC scales. Two ideas underline this result: one related to measure design and another linked to evaluated aspects. Firstly, KPST might be more willing to complete simple and graphic scales, such as the INS (Martin and Czellar, 2016), hence the drawing representation of *nature* and *self* might facilitate them to consider how much connected to the environment they feel, and such connection significantly increased. Secondly, in comparison to LCN and NR-6, INS and CCC measure more cognitive than emotional and attitudinal aspects; and significant differences were only shown for the cognitive dimension. This fact might indicate that, after the experience in the OLG, participants were more conscious on their connection to the natural environment but not more affectively connected.

The significant differences found in quantitative measures relate somehow with the *conceptions of nature* hold by KPST. The predominant initial *conception of nature* was mainly static, simplistic, non-social, and biased toward the most obvious natural elements. These results are similar to those reported in previous studies with students from different educational levels (Loughland et al., 2002; Payne et al., 2014), including KPST (Flogaitis and Agelidou, 2003). Similarly, previous research indicates that most students encountered difficulties with understanding nature as a complex and dynamic Earth system (Eilam, 2012; Sharma and Buxton, 2018). In our study, a previously reported tendency of students to exclude humans from their conception of the natural world (Shepardson et al., 2007; Li and Ernst, 2015) was also identified. The existence of a conceptual dichotomy between “nature” and “culture,” also named “human–nature binary,” has been widely discussed and considered characteristic of a western worldview in the modern era (Castree, 2013), but not universal (Descola, 2013). This is closely related to the *utilitarian* view that was shown by part of the students, which had also been identified in Sharma and Buxton’s (2018) research. Nevertheless, Li and Ernst (2015) reported that students could simultaneously show the inclination to protect and treat nature with respect, as it was the case for the students in C8.

After the garden experience, changes occurred mostly from conceptions included in the groups *static non-pristine* (C3–C5) and *dynamic only human–nature interactions* (C6–C8), in such a way that the number of definitions allocated into C9 (*Systemic*) increased from 7 to 19% of the total students. Relevant concepts of Ecology and Environmental Sciences, such as “cycles,” “ecosystem,” “global,” “interdependence,” or “biodiversity” appeared in the final KPTS’ definitions of nature, in line with previous findings on the use of UOLGs in Education for Sustainability (Eugenio et al., 2018). Albeit this increase, when the whole category system was quantitatively evaluated, non-statistically significant differences appeared.

## Conclusion

Overall, KPST showed connection to nature, more related to cognitive than to emotional or attitudinal aspects, and their conceptions of nature seem to be predominantly simplistic. This might indicate that even though participants reported to be connected to nature, their idea about nature does not include a complex relation between all the living and non-living things and the processes and interactions occurring. Considering this idea, it seems that more research in OLG should be done to promote attachment, care, and love toward the environment, since attitudinal and emotional aspects of the environment link to pro-environmental behaviors (Schultz, 2001; Zelenski et al., 2015). From the point of view of science education, it is relevant to promote the evolution of students’ *conceptions of nature* toward a global, systemic, and multidimensional view. Flogaitis and Agelidou (2003) found that environmental education programs were not able to influence KPST’s *conceptions of nature*. It is known that learners’ conceptions are rooted systems of persistent ideas, which result difficult to change and thus remain even through different educational stages, coexisting in children and young of varying ages (Taber, 2015). Further efforts to better teach the complex, comprehensive, and macroscale aspects of the nature concept are undoubtedly necessary and valuable, particularly for initial teacher training. Additionally, research in the area should be conducted to understand the intricate relations between experience the natural environment and the connectedness and conception of nature.

## Data Availability Statement

The datasets generated for this study are available on request to the corresponding author.

## Ethics Statement

Ethical review and approval was not required for the study on human participants in accordance with the local legislation and institutional requirements. The participants provided their written informed consent to participate in this study.

## Author Contributions

ME-G and RP-L designed the study. Data were collected by ME-G, RP-L, and DZ. The data were analyzed by RP-L, DZ, and AR-G. All authors wrote the manuscript.

## Conflict of Interest

The authors declare that the research was conducted in the absence of any commercial or financial relationships that could be construed as a potential conflict of interest.

## Footnotes

1 CCC’s items: Item 1, *Climate change is real*; Item 2, *Climate change impacts my daily life*; and Item 3, *Usually, I behave pro-environmentally*.
