# Supplementary material for: Organic Learning Gardens in Higher Education: Do They Improve Kindergarten Pre-service Teachers’ Connectedness to and Conception of Nature?
Source: Front Psychol. 2020 Mar 10;11:282. doi: 10.3389/fpsyg.2020.00282 (PMC7076075; doi:10.3389/fpsyg.2020.00282)
Supplement: Supplementary file 1 [file Data_Sheet_1.PDF]

frontiers

Supplementary Material

Supplementary Material for the paper: Organic Learning Gardens in Higher Education: do they improve kindergarten pre-service teachers' connectedness to and conception of nature?

Detailed description of: 1) the phenomenographic analysis that was conducted in this study, and 2) each of the nine qualitatively different KPST's conceptions of nature that were unveiled

1. Phenomenographic analysis:

Phenomenography is a qualitative research approach which aims to investigate and describe the full range of ways in which people think about or experience a concept or phenomenon (Marton, 1981 1994, 2015). It deals with how the range of ways of perceiving and understanding reality can be classified into a system of hierarchic categories of growing complexity. In this study, the range of conceptions of nature given by kindergarten pre-service teachers (KPST) is enclosed into a hierarchic series of descriptive categories, following the criteria of Marton and Booth (1997). Such categories were constructed basing on students' responses themselves, and the system of categories intends to reflect an increasing level of understanding and experiencing what nature is. In order to achieve it, one researcher performed a preliminary analysis on a subsample of 10% of the definitions of nature given by students, and grouped them into categories. Independently, two different researchers performed an analysis on the totality of definitions and drafted a list of outstanding conceptions. By contrasting both analyses, an initial system of categories was established, afterwards it was reviewed and researchers discussed it until they reached a consensus on a final hierarchic system of categories. Independently, two researchers evaluated again students' definitions of nature using the initial system of categories in order to determine if defined categories were sufficiently descriptive and covered the full range of evidenced views (Marton & Booth, 1997). Afterwards, both researchers compared and exhaustively discussed each definition allocation, in order to achieve trustworthiness (Cohen's Kappa reliability coefficient average 0.92). Thus, the refined final definition of the system of categories used to analyse students' conceptions of nature was obtained after an iterative process of comparison, discussion, and re-definition to consensus.

2. Categories of conceptions of nature that were unveiled (please check Table 2):

Static conceptions of nature:

In the first five conceptions (C1 to C5), nature is understood or experienced by KPST as a static entity. All of them correspond to simplistic views of nature, which is mainly defined as a 'place', and characterized by means of a quite restricted list of wide categories of living organisms and/or physical components. Hence, in these categories, the biological and/or physical dimensions of nature are referred to, and nature is described basing on its most prominent elements. Students expressing static conceptions take one of the following two ideological stances: either nature is explicitly unspoiled (static pristine conceptions of nature: C1 and C2), or this aspect is not explicitly acknowledged (static non pristine conceptions: C3 and C4).

In C1 and C2 categories, nature is believed to be ‘out there’ in a pure, untouched state (‘everything that the human being has not modified and that has been given to us naturall’, ‘a zone that is not built or manipulated by the human being’). Moreover, definitions in these categories include descriptions based on elements of the biophysical world (C1-’everything around us’, ‘the place around us’, ‘where we live’, ‘all things that exists’, ‘all the living things’; C2 -’ a set of living and non-living things’, ‘animals, plants, rocks and mountains’, ‘biotic and abiotic elements’, ‘our landscape, rivers, trees, plants and animals’). The difference between C1 and C2 categories is that C2 encloses conceptions which describe nature as static and pristine, but moreover mention both biological and physical elements. Categories C3 and C4 are analogous to C1 and C2, respectively, and exclusively differ from them because these conceptions do not make explicit a pristine view of nature.

Finally, a more informed static view is enclosed in C5 category (Biophysical diverse): these conceptions provide with some ‘ecological’ insights, in the sense that they acknowledge the existence of change and diversity in nature (e.g. ‘nature can have topographic and climatological differences that allow the life of different living being’, ‘depending on their climatic characteristics, some plants or others will grow better’, ‘there is not the same nature in Northern that in Southern Spain, due to climate, vegetation, pollution, type of terrain, rocks, etc.’, ‘in nature you can find different elements, ecosystems and animals depending on the area in which you are’).

Dynamic conceptions of nature:

The next four conceptions (C6 to C9) are qualitatively different since nature is considered as a dynamic entity. Conceptions enclosed into categories C6 to C8 describe nature as a place characterized by biophysical elements – similarly to C1-C6 – but moreover emphasize the existence of dynamic interactions between humans and the natural world. In these three categories, most KPST include a value-orientation to their conception of nature.

Thus, conception C6 is well aligned to the belief that humans have a relationship with nature which is primarily utilitarian, focused on how nature supports human’s life quality by providing us with goods and services. At the same time, it involves the recognition of human physical dependence on natural systems. KPST express it as: ‘thanks to nature human beings have a good existence because it partly influences our development’, ‘It is essential for human beings, both for their health and for food’, ‘helps us to survive’, ‘we obtain from nature vegetables, meat, fish and all food that we take come from the earth, are alive or are made with products of this type’, ‘nature provide us all the resources we need to live’.

The value-orientation of conception C7 is the belief that humans are ‘stewards’ of nature, and thus responsible for the maintenance of a livable environment; the focus is preservation. Thus, these understandings are related to the moralistic value that involves the recognition of an ethical responsibility to treat nature with respect and to protect it, for instance: ‘it is very important to take care of and respect it’, ‘We have to take care of it and not contaminate it, because if you harm it, it affects you too’.

Conception C8 integrates together the views of nature already described for C7 and C8: nature is at the same time something that we must preserve and protect for it is necessary to support our life’s quality (utilitarian + preservation): ‘nature is an important source of products, so we must take care of it’, ‘the respect to and care of the natural world is fundamental for human evolution and for the planet in general’.

Finally, the most informed conception that was unveiled among KPST is enclosed in C9. This expands the dynamic view of nature through a focus not only on human to nature relationships, but also on interactions that occur between different earth systems (systemic). Thus, it integrates some notions regarding interdependence and/or cyclic nature of natural phenomena, and it is at least partially systemic and complex. In these definitions of nature, relevant concepts of Ecology and Environmental Sciences, such as 'cycles', 'ecosystem', 'global', 'interdependence', or 'biodiversity' are mentioned, which are more aligned with contemporary scientific conceptions about nature and its functioning. Some examples of statements that were enclosed into this category are: 'It is made up of ecosystems, fauna, flora, living organisms, landscapes and human beings. All of them are part of a global set that interrelates', 'The importance of how living beings are related to the environment is fundamental to know the functioning and characteristics of the natural environment', 'Nature is the set of ecosystems that surrounds us. It is studied through natural sciences and this includes other sciences such as geography, geology, biology, physics and astronomy, etc.', 'is an interrelated environment, with a set of living beings with specific behaviors that interact with each other in complex ways', 'It is a system of interrelated elements in which the elements are living beings and their habitat and different processes and interactions'

References

Marton, F. (1981). Phenomenography—describing conceptions of the world around us. Instructional science, 10(2), 177-200.

Marton, F. (1994). Phenomenography. In T. Husén, G. Handal, & T. N. Postlethwaite (Eds.), The international encyclopedia of education (2nd ed., pp. 4424–4429). Oxford, UK: Pergamon Press.

Marton, F. (2015). Necessary conditions of learning. New York, NY: Routledge

Marton, F. & Booth, S. (1997). Learning and awareness. London: Routledge.
